# 

**Published:** 1906-07-07

**Authors:** 


					July 7th 190G.
*FALf
?W.*.*uurr ntSTEHH INFIKMAnr
No. 1,03? Vol. XL [??S??] Saturday,
&
ulyTth, 190c
,bHCeeippti2056Tern}S] PRICE THREEPENCE

				

